# The effects of intro-oral parathyroid hormone on the healing of tooth extraction socket: an experimental study on hyperglycemic rats

**DOI:** 10.1590/1678-7757-2019-0690

**Published:** 2020-04-27

**Authors:** Lin XU, Li MEI, Rui ZHAO, Jianru YI, Yixuan JIANG, Ruomei LI, Youliang ZHAO, Li PI, Yu LI

**Affiliations:** 1 National Clinical Research Center for Oral Diseases West China Hospital of Stomatology Sichuan University Chengdu China State Key Laboratory of Oral Diseases, National Clinical Research Center for Oral Diseases, Department of Orthodontics, West China Hospital of Stomatology, Sichuan University, Chengdu, China.; 2 Sir John Walsh Research Institute Faculty of Dentistry University of Otago New Zealand Discipline of Orthodontics, Department of Oral Sciences, Sir John Walsh Research Institute, Faculty of Dentistry, University of Otago, New Zealand.; 3 National Clinical Research Center for Oral Diseases West China Hospital of Stomatology Sichuan University Chengdu China State Key Laboratory of Oral Diseases, National Clinical Research Center for Oral Diseases, Department of Maxillofacial Surgery, West China Hospital of Stomatology, Sichuan University, Chengdu, China.; 4 National Clinical Research Center for Oral Diseases West China Hospital of Stomatology Sichuan University Chengdu China State Key Laboratory of Oral Diseases, National Clinical Research Center for Oral Diseases, Department of Oral Implantology, West China Hospital of Stomatology, Sichuan University, Chengdu, China.; 5 Ninth People’s Hospital School of Stomatology Shanghai Jiao Tong University Shanghai China Department of Orthodontics, Ninth People’s Hospital, School of Stomatology, Shanghai Key Laboratory of Stomatology, Shanghai Jiao Tong University, Shanghai, China; 6 Department of emergency department West China Second Hospital Sichuan University Chengdu China Department of emergency department, West China Second Hospital, Sichuan University, Chengdu, China; 7 West China School of Public Health Sichuan University Chengdu China West China School of Public Health, Sichuan University, Chengdu, China

**Keywords:** Diabetes mellitus, Tooth extraction, Parathyroid hormone, Wound healing

## Abstract

**Objective:**

To investigate the effects of intro-oral injection of parathyroid hormone (PTH) on tooth extraction wound healing in hyperglycemic rats.

**Methodology:**

60 male Sprague-Dawley rats were randomly divided into the normal group (n=30) and DM group (n=30). Type 1 diabetes mellitus (DM) was induced by streptozotocin. After extracting the left first molar of all rats, each group was further divided into 3 subgroups (n=10 per subgroup), receiving the administration of intermittent PTH, continuous PTH and saline (control), respectively. The intermittent-PTH group received intra-oral injection of PTH three times per week for two weeks. A thermosensitive controlled-release hydrogel was synthesized for continuous-PTH administration. The serum chemistry was determined to evaluate the systemic condition. All animals were sacrificed after 14 days. Micro-computed tomography (Micro-CT) and histological analyses were used to evaluate the healing of extraction sockets.

**Results:**

The level of serum glucose in the DM groups was significantly higher than that in the non-DM groups (p<0.05); the level of serum calcium was similar in all groups (p>0.05). Micro-CT analysis showed that the DM group had a significantly lower alveolar bone trabecular number (Tb.N) and higher trabecular separation (Tb.Sp) than the normal group (p<0.05). The histological analyses showed that no significant difference in the amount of new bone (hard tissue) formation was found between the PTH and non-PTH groups (p>0.05).

**Conclusions:**

Bone formation in the extraction socket of the type 1 diabetic rats was reduced. PTH did not improve the healing of hard and soft tissues. The different PTH administration regimes (continuous vs. intermittent) had similar effect on tissue healing. These results demonstrated that the metabolic characteristics of the hyperglycemic rats produced a condition that was unable to respond to PTH treatment.

## Introduction

Diabetes mellitus (DM) has an increasingly higher occurrence of 463 million adults worldwide.^1^ People with DM are more likely to suffer from earlier detrimental oral status and are prone to numerous oral diseases, including oral infection, periodontitis and difficulties in wound healing after tooth extraction.^2^ It has been found that the prevalence of tooth extractions in DM patients is 1.88 times higher than the general population.^3^

The high-glucose microenvironment would delay the healing of tooth extraction sockets.^4^ High glucose could affect the osteoblastic function and matrix mineralization.^5^ The soft tissue repair is associated with the defect in the formation of granulation tissue and collagen degradation induced by excessive matrix metalloproteinase.^6^ The dysfunction of fibroblast could impair the collagen synthesis, resulting in decreased fiber accumulation.^7^ Many studies have explored different approaches to accelerate the healing of tooth extraction sockets under DM conditions, such as the use of growth factors,^8^ graft fillings,^9^ ellagic acid,^10^ and low-level laser therapy.^11^ The effectiveness of these approaches, however, is unsatisfactory due to the complexity of DM and vulnerability of the oral environment.^12^

Parathyroid hormone (PTH) is one of the important hormones to maintain the balance of the calcium and phosphorus metabolism.^13^ PTH can regulate osteoclast activity and influence bone remodeling, and is currently the only clinical drug to promote osteogenesis for the treatment of osteoporosis.^14^ The intermittent administration of PTH has been found to directly inhibit transcription of the sclerosis gene and stimulate bone formation;^15^ the continuous administration of PTH could increase the receptor activator of the nuclear factor-kB ligand/osteoprotegerin ratio, resulting in an increased osteoclastogenesis.^16^ PTH could also benefit the healing of soft tissue via suppressing inflammation and stimulating collagen deposition.^4^ A recent study has reported that PTH promoted the healing of both hard and soft tissues in the tooth extraction socket.^17^

The effect of PTH on extraction socket healing under DM condition remains unclear. The aim of this study was to investigate and compare the effects of local administration (intro-oral injection) of intermittent- and continuous-PTH on the healing of tooth extraction sockets in hyperglycemic rats.

## Methodology

### Synthesis and characterization of PTH controlled-release hydrogel

A thermosensitive and injectable hydrogel was prepared for the continuous administration of PTH based on the literature.^18^ The poly (ethylene glycol)-poly (caprolactone)-poly (ethylene glycol) thermosensitive hydrogel (PEG-PCL-PEG, PECE) was synthesized by ring-opening copolymerization.^19^ The ^1^H Nuclear Magnetic Resonance (^1^H NMR) spectra (in CDCl^3^ ) was recorded on a Varian 400 spectrometer (Varian, USA) at 400 MHz to characterize chemical composition of PECE copolymers.

The sol–gel–sol phase transition behavior was carried out as follows: PECE hydrogel was placed into 5 mL EP tube and incubated in a water bath at 0°C for ten min, and then was slowly heated at a rate of 0.5°C/min until 37°C. The sol–gel–sol phase transition diagram was recorded using the test tube-inverting method, which was visually observed by inverting the tube. The condition of sol and gel phase was defined as “flow liquid sol” and “no flow solid gel”.

*In vitro* release tests at body temperature were carried out as follows: firstly, 750 μL hydrogel loaded with 1 mg BAS protein was placed into the bottom of the EP tube at 37°C for 1 h to form the gel. Then, 1000 μL Milli-Q water was added into the tube to immerse the gel. Then, the sample was kept and shaken at 106 rpm at 37°C. The solution was collected at each time of interest (T=0,1, 2, 4, 8, 12, 24 h in the first day, and at each 24 h interval until 336 h) and replaced with 1000 μL preheated fresh Milli-Q water. After centrifugation at 13,000 rpm for 10 min, the measured extracts were stored at −20°C for drug release analysis. The amount of released protein at each time points was assessed in triplicate using the Enhanced BCA Protein Assay kit (Beyotime technology, Beijing, China) following manufacturer’s instructions. The accumulative release percent of BSA protein was calculated.

### Animal model and tooth extraction

A total of 60 young male Sprague-Dawley rats (5 weeks old) were included in the study. Sample size was calculated by power analysis based on the estimated effect size reported in the previous study.^20^ Rats were housed at 22 ºC, 40% humidity, and in 12-hour daylight cycles. Rats were allowed access to standard laboratory rodent diet and water. The ethics of the study was approved by the Research Ethics Committee of West China Hospital of Stomatology (No. 201610610379). The study was presented following the Animal Research N3CRs guidelines for Reporting of In Vivo Experiments (ARRIVE) guidelines.

Sixty rats were randomly divided into two groups (n=30 per group), including the normal control and the diabetic group. Diabetes was induced into 12h-fasted rats by intraperitoneally streptozotocin (STZ) (Sigma, St Louis, MO, USA) dissolved in citrate buffer (pH=4.2-4.5). STZ was calculated at 45 mg/kg.^21^ Three days after the STZ injection, blood was obtained from the tail vein, and glucose levels were measured using a glucometer (Yuwell, Jiangsu, China). Diabetes was confirmed twice by the presence of random blood glucose concentrations of >16.7 mmol/L.^10^

The mandibular left first molar was extracted under general anesthesia (10% chloral hydrate solution, 0.32ml/100g) from each animal. Sterile dental instruments were used during the extraction of molars, and teeth removal was performed by using a sharpened dental explorer.^22^ Alveolar sockets were left to heal without sutures.

### Grouping and administration of PTH

After tooth extraction, the two groups were divided randomly into 3 subgroups (n=10 per group), including the normal saline (NS), the intermittent-PTH (iPTH) and the continuous-PTH group (cPTH).

The intermittent intra-oral injection of PTH (1-34) (Chinese Peptide, Hangzhou, China) was administered at a dose of 80 μg/kg three times a week for 14 days.^17 , 23^ It was dissolved in normal saline solution, and the needle was placed in the buccal vestibule next to the tooth extraction site.

The continuous intra-oral injection of PTH (1-34) was administered as follows: PECE was mixed and dissolved completely in NS to form sol in the temperature of 55-60ºC at a concentration of 25 wt% and cooled at 0 ºC for three minutes.^19^ Then, PTH (1-34) solution was mixed into the PECE sol to form suspension, and the PTH (1-34) gel to release slowly for 14 days was obtained. Soon after the tooth extraction, PECE hydrogel loaded with an equivalent dosage of PTH (1-34) in iPTH group was put in the tooth socket.

Animals in the two NS groups (NS normal group and NS diabetic group) were injected with the same volume of normal saline solution three times a week for 14 days.

### Serum chemistry

Blood was collected from carotid artery at the time of euthanasia, which was 24 h after the last injection of PTH. Serum samples were prepared and used immediately. The levels of calcium were measured using a commercial kit (Jiancheng, Nanjing, China). The blood glucose levels from the tail vein of each group were monitored once every week using a glucometer (Yuwell, Jiangsu, China). Diabetic rats with unsatisfactory serum glucose levels would be removed; however, no animal was excluded for this reason.

### Micro computed tomography (Micro-CT) assessment

Rats were euthanized at the end of the designated injection period of 14 days. The mandibles were dissected, and fixed in 10% formalin, and the extraction wounds were scanned by Micro-CT (Micro-CT50, Scanco Medical, Bassersdorf, Switzerland) at 10 μm voxel resolution with the beam energy level of 70 kVp and electrical current of 0.2 mA. The time of scanning was 858 ms. Each mandible was fixed by upside-down styrofoam, and the occlusal plane became horizontal to the bottom. The conservation of the samples occured in 10% formalin. The thresholds were determined visually in several slices. Trabecular parameters were identified by the direct measurement technique. The region of interest was a trabecular compartment of 100 slices around extraction sockets and was segmented by semi-manual contour. Alveolar bone trabecular number (Tb.N), trabecular thickness (Tb.Th), trabecular separation (Tb.Sp) and bone mineral density (BMD) were analyzed with built-in software.

### Histomorphometric analysis

Rats were decapitated two weeks after the interventions. The specimens were demineralized in 10% ethanol, paraffin-embedded, and sectioned to 5 μm thickness. Hematoxylin and eosin (HE) (Beyotime technology, Beijing, China) and Masson’s trichrome (Sigma, St Louis, MO, USA) staining techniques were performed for the detection of bone tissue and collagen fibers respectively, following the manufacturer’s instructions. The staining sections were analyzed by Image-Pro Plus (Media Cybernetics, Bethesda, MD, USA). New alveolar bone formation was quantified in the extraction wounds as described previously.^20^

### Statistics analysis

Statistical analyses were performed using the SPSS statistics base 17 (SPSS Inc., Chicago, IL, USA). Results are presented as mean±standard deviation. The normality test was performed using the Shapiro-Wilk analysis; the statistical comparisons were analyzed by the Student’s t-test, one-way ANOVA, and two-way ANOVA, with a significance level of 5%. Statistical significance was defined as 0.05 α-level.

## Results

### Characterization of PTH controlled-release hydrogel

**Figure 1:**
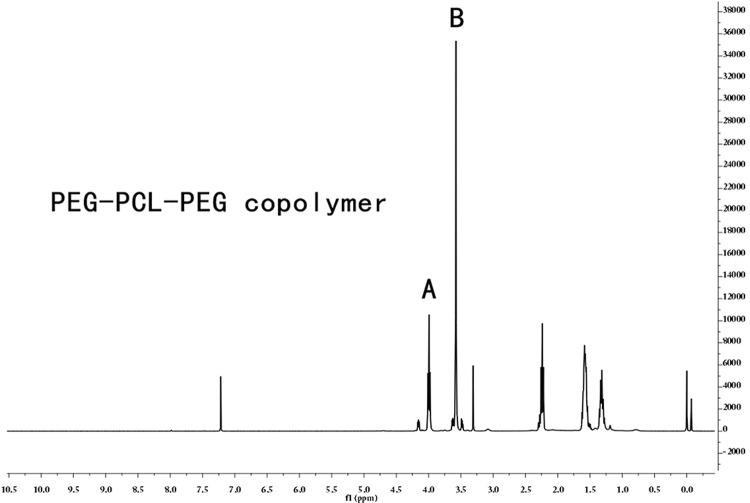
1H NMR spectrum of PECE copolymer. The peak of (A) caprolactone unit and (B) ethtlene glycol unit were at 3.98ppm and 3.57ppm, respectively

The chemical structure of PECE gel was characterized by ^1^H NMR ( Figure 1 ). The peak of the caprolactone unit and the ethylene glycol unit were at 3.98 ppm and 3.57 ppm, respectively. The macromolecular weight estimated from ^1^H NMR spectrum was 3246, which was consistent with theoretical estimation, indicating that the PECE gel was synthesized successfully.

**Figure 2:**
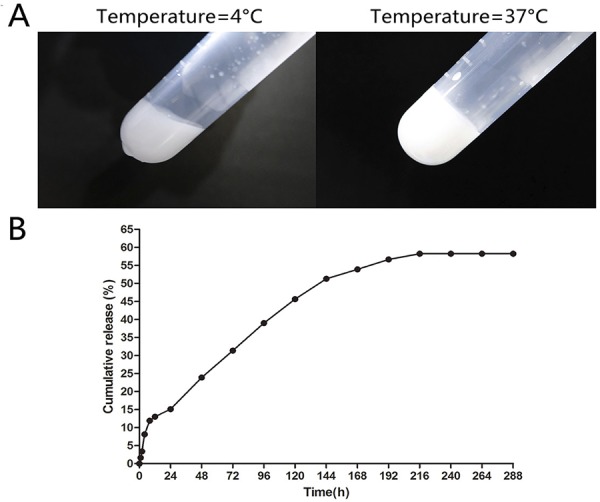
Characterization of PTH controlled-release hydrogel (A) The sol-gel phase transition behavior of PECE hydrogel: the solution state of hydrogel at 0°C and the gel state of hydrogel at 37 °C. (B) The cumulative release of drug-loaded PECE hydrogel for 14 days

The temperature-dependent sol–gel phase transition behavior was presented in Figure 2A . The hydrogel showed a sol state at lower temperature (0°C) and a gel state at body temperature (37°C). The hydrogel underwent sol-gel-sol phase transition as the temperature increased and slowly released PTH at local part, which prolonged the therapeutic effect time and reduced the repeated injections.

The controlled-release ability of PECE hydrogel is shown in Figure 2B . The release rate of the drug was rapid in the first 168 hours, and then became comparatively slow and sustained in the next 168 hours. The cumulative release of protein reached 58.24% by the 14th day. Therefore, the experiment period for the PTH controlled-release hydrogel was set as 14 days in this study.

### Serum chemistry

The level of serum glucose in the DM groups (DM+NS, DM+iPTH and DM+cPTH) (mean 22.38±2.71 mmol/L, with no significant difference within the three groups, p>0.05) was significantly higher than the non-DM groups (NOR+NS, NOR+iPTH and NOR+cPTH), (mean 5.82±0.67 mmol/L) (p<0.01), indicating that the DM model was successfully established in the study.

No statistical significance of the serum calcium concentration was found either between the DM and non-DM groups, or within these groups (p>0.05 for all). Local administration in the extraction socket in the study could reduce the systematic effects of PTH, such as hypercalcemia, which was reflected by the unaltered serum calcium level.

### Micro-CT analysis of the extraction socket

**Figure 3:**
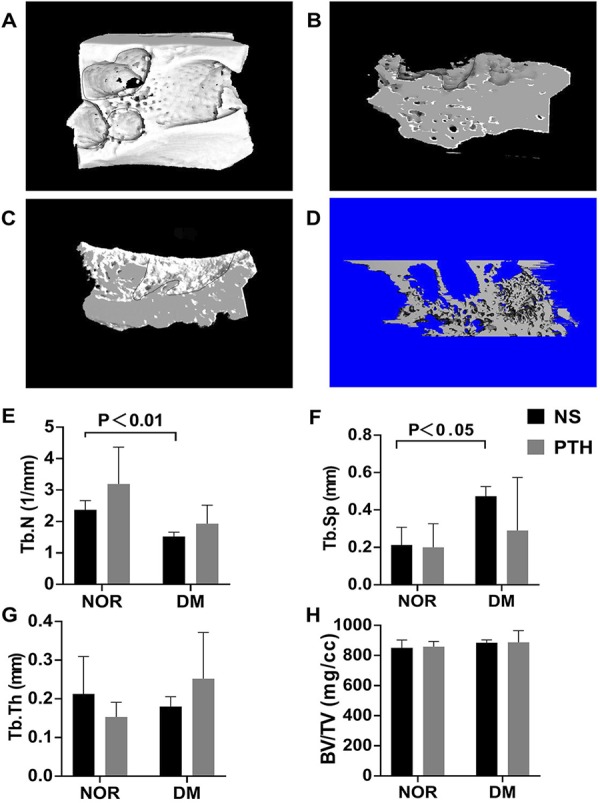
Micro-CT analysis of the extraction socket. (A) Cross section of the Micro-CT scan in the extraction socket; (B) Sagittal plane of the Micro-CT scan in the DM+iPTH group; (C) Sagittal plane of the Micro-CT scan in DM+NS group; (D) 3D reconstruction of the extraction socket in DM+PTH group. (E), (F), (G) and (H) Micro-CT analysis of Tb.N, Tb.Sp, Tb.Th and BMD. Tb.N in NOR+NS group was significantly higher than that in DM+NS group (p<0.01), while the Tb.Sp in NOR+NS group was significantly lower than that in DM+NS group (p<0.05). PTH therapy for 14 days induced no detectable bone anabolic effect in alveolar socket bone parameters

The Micro-CT analysis ( Figure 3 ) showed that the Tb.N in NOR+NS group (2.37 ± 0.29 mm^-1^) was significantly higher than that in DM+NS group (1.52 ± 0.14 mm^-1^) (p<0.01) ( Figure 1E ), while the Tb.Sp in NOR+NS group (0.21 ± 0.09 mm) was significantly lower than that in DM+NS group (0.47±0.05 mm) (p<0.05) ( Figure 1F ), indicating that bone density was affected by the DM condition. No significant difference was found among all the other groups for the alveolar bone Tb.Th and BMD (p>0.05 for all). Bone formation in the extraction socket under the DM conditions in the study was significantly less than that under non-DM condition, indicating an impaired hard-tissue healing of extraction socket under DM condition.

There is no significant difference of Tb.N, Tb.Th, Tb.Sp and BMD between the PTH and NS groups (p>0.05 for all), indicating that the effect of PTH on tooth extraction socket in normal and DM rats was minimal ( Figure 2 E-H ).

### Histological analysis of the extraction socket

**Figure 4:**
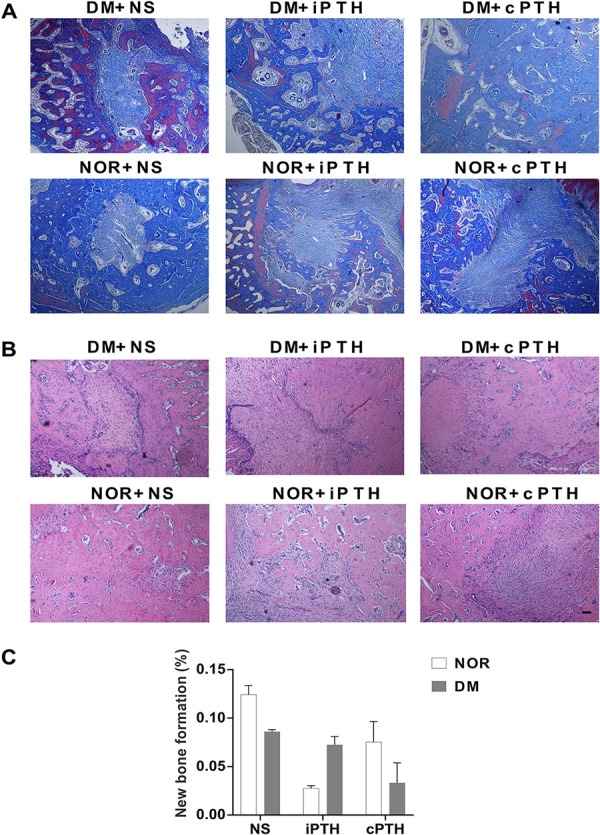
Histological analysis of the extraction socket. (A) Masson’s trichrome staining (red parts meant new bone and light blue parts meant collagen. Scale bar: 100μm); (B) HE staining; and (C) New alveolar bone formation in the tooth extraction socket. PTH therapy for 14 days induced no detectable new alveolar bone formation

The histological analysis of soft tissue (HE and Masson’s trichrome staining) showed that more collagen in the extraction socket was observed in the groups treated with PTH (both iPTH and cPTH) than those not treated with PTH ( Figure 4A and B ). The quantitative analysis of hard tissue (new alveolar bone formation) showed no statistical significance between DM and non-DM groups or between NS and PTH groups (p>0.05) ( Figure 4C ).

## Discussion

In this study, bone formation in the extraction socket in the DM rats was decreased when compared to that of the normal control rats. PTH (1-34) showed no obvious results in hard tissue formation and soft tissue healing. No difference was found on the healing of extraction socket between intermittent and continuous administration of PTH. These findings suggest that hyperglycemic rats failed to benefit from the anabolic treatment of PTH on the healing of extraction socket in the high glucose condition.

The effectiveness of PTH on promoting the healing of tooth extractions in the diabetic rats was not significant in the study. This may be due to the metabolic characteristics of hyperglycemic rats leading to a condition of ineffective PTH treatment. Some studies showed that PTH did not increase the insulin-immunoreactivity of β-cells of the pancreatic islets in diabetic rats.^24^ The inflammation caused by diabetes would disrupt vascular endothelial cell function.^25^ The expression of inflammatory cytokines such as TNF-α, IL-1 and IFN-γ in bone increased,^26 , 27^ which contributed to the delayed healing of extraction sockets. Moreover, the high level of advanced glycosylation end products and collagen cross-linking may cause an overall negative effect on diabetic bone mechanics.^28^ Therefore, metabolic control of hyperglycemia may be a key factor when patients with diabetes receive anabolic therapy to improve bone mass. Future studies could explore the anabolic property of PTH in the DM condition when hyperglycemia is controlled with insulin, and immunolabeling or molecular analyses could be performed to further evaluate the possible anabolic activity of PTH and signaling pathways.

The histological analyses in the study showed that the PTH treatment showing more collagen formation demonstrated an anabolic effect on soft tissue healing. In response to hyperglycemia, advanced glycosylation end products were induced and covalently bounded to reactive amino groups, resulting in a dysfunction of the epithelial cells and extracellular matrix.^7^ This is coincident with the decreased collagen in the extraction socket in the DM rats. This may be due to the microcirculation defect and insufficient oxygen caused by thickened and inelastic vessel wall.^29^

Several studies have shown double effects of PTH (1-34) – anabolic when administered intermittently and catabolic when applied continuously.^30^ The subcutaneous and intermittent administration of PTH facilitated the hard and soft tissue repair in tooth extract socket at the dose of 80 μg/kg daily.^4^ Intermittent and systemic PTH administration reduced alveolar bone loss by decreasing the receptor activator of nuclear factor kappa B ligand (RANKL)/osteoprotegerin (OPG) ratio in hyperglycemic rats.^23^ However, in the continuous administration of PTH, the expression of Phospho1 and Smpd3 in osteoblasts decreased,^16^ resulting in the suppression of bone formation mediated by Cox2.^31^

Due to the biological nature of hard-tissue healing in the tooth extraction socket,^32^ this study mainly focused on the effects of PTH on bone formation rather than resorption. However, hyperglycemic rats failed to benefit from the anabolic treatment. The contradictory action of PTH might be caused by double effect on bone formation and bone resorption. The anabolic role of iPTH could be lessened by the catabolic effect of DM.^33^ At the early stage of bone and cartilage healing process, both administrations of PTH could promote osteogenic differentiation.^34^ The cPTH did not suppress osteogenesis, but the catabolic role on bone metabolism was greater than its anabolic role, resulting in bone loss. The cPTH increased slightly undifferentiated mesenchymal stem cell, osteoblast and osteocyte number at 14 days.^35^ This may result in the non-significant difference between two regimes of PTH. Another possible reason might be that the rats used in the study were male. The cPTH was found to be not catabolic in male rats, as no change was found in BMD and cortical thickness in males.^36^

The experiment period adopted in this study was two weeks. This was in consideration of the optimal slow-release effect of the PECE hydrogel. Furthermore, the epithelialization was generally complete and well-keratinized at approximately two weeks.^37 , 38^ The maximum bone formation was achieved and the woven bone completely filled the extraction socket,^4^ and almost replaced by substance with a radio-opacity during that timeframe,^37^ so 14 days was appropriate for this study. The long-term effects of PTH on the healing of extraction socket under hyperglycemic conditions need further investigation.

## Conclusion

Bone formation in the extraction socket under the DM condition was reduced. Hyperglycemic rats failed to benefit from the PTH treatment in the healing of tooth extraction sockets. The different PTH administration regimes (intermittent *vs.* continuous) had similar effects on the tissues healing.

## References

[1] - International Diabetes Federation. IDF Diabetes Atlas. 9th ed. Brussels: IDF; 2019.

[2] - Barasch A, Safford MM, Litaker MS, Gilbert GH. Risk factors for oral postoperative infection in patients with diabetes. Spec Care Dentist. 2008;28(4):159-66. 10.1111/j.1754-4505.2008.00035.x 18647376

[3] - Mayard-Pons ML, Rilliard F, Libersa JC, Musset AM, Farge P. Database analysis of a French type 2 diabetic population shows a specific age pattern of tooth extractions and correlates health care utilization. J Diabetes Complications. 2015;29(8):993-7. 10.1016/j.jdiacomp.2015.09.007 26463898

[4] - Kuroshima S, Mecano RB, Tanoue R, Koi K, Yamashita J. Distinctive tooth-extraction socket healing: bisphosphonate versus parathyroid hormone therapy. J Periodontol. 2014;85(1):24-33. 10.1902/jop.2013.130094 PMC4245052 23688101

[5] - Lozano D, Castro L, Dapía S, Andrade-Zapata I, Manzarbeitia F, Alvarez-Arroyo M, et al. Role of parathyroid hormone-related protein in the decreased osteoblast function in diabetes-related osteopenia. Endocrinology. 2009;150(5):2027-35. 10.1210/en.2008-1108 19196804

[6] 6 - Zhang Y, McClain S, Lee H, Elburki M, Yu H, Gu Y, et al. A novel chemically modified curcumin “normalizes” wound-healing in rats with experimentally induced type i diabetes: initial studies. J Diabetes Res [Internet]. 2016 [cited 2020 Mar 2];2016:5782904. Available at: 10.1155/2016/5782904 PMC4846750 27190999

[7] - Lioupis C. Effects of diabetes mellitus on wound healing: an update. J Wound Care. 2005;14(2):84-6. 10.12968/jowc.2005.14.2.26738 15739657

[8] - Mozzati M, Gallesio G, di Romana S, Bergamasco L, Pol R. Efficacy of plasma-rich growth factor in the healing of postextraction sockets in patients affected by insulin-dependent diabetes mellitus. J Oral Maxillofac Surg. 2014;72(3):456-62. 10.1016/j.joms.2013.10.010 24342581

[9] - Martínez-Santamaría L, Conti C, Llames S, García E, Retamosa L, Holguín A, et al. The regenerative potential of fibroblasts in a new diabetes-induced delayed humanised wound healing model. Exp Dermatol. 2013;22(3):195-201. 10.1111/exd.12097 23489422

[10] 10 - Al-Obaidi MM, Al-Bayaty FH, Al Batran R, Hussaini J, Khor GH. Impact of ellagic acid in bone formation after tooth extraction: an experimental study on diabetic rats. ScientificWorldJournal [Internet]. 2014 [cited 2020 Mar 2];2014:908098. Available at: 10.1155/2014/908098 PMC4251085 25485304

[11] - Park JJ, Kang KL. Effect of 980-nm GaAlAs diode laser irradiation on healing of extraction sockets in streptozotocin-induced diabetic rats: a pilot study. Lasers Med Sci. 2012;27(1):223-30. 10.1007/s10103-011-0944-8 21732114

[12] - Chandu A, Macisaac RJ, Smith AC, Bach LA. Diabetic ketoacidosis secondary to dento-alveolar infection. Int J Oral Maxillofac Surg. 2002;31(1):57-9. 10.1054/ijom.2001.0140 11936401

[13] - Silva BC, Bilezikian JP. Parathyroid hormone: anabolic and catabolic actions on the skeleton. Curr Opin Pharmacol. 2015;22:41-50. 10.1016/j.coph.2015.03.005 PMC5407089 25854704

[14] - Tashjian AH Jr, Chabner B. Commentary on clinical safety of recombinant human parathyroid hormone 1-34 in the treatment of osteoporosis in men and postmenopausal women. J Bone Miner Res. 2002;17(7):1151-61. 10.1359/jbmr.2002.17.7.1151 12096828

[15] - Keller H, Kneissel M. SOST is a target gene for PTH in bone. Bone. 2005;37(2):148-58. 10.1016/j.bone.2005.03.018 15946907

[16] - Houston D, Myers K, MacRae V, Staines K, Farquharson C. The expression of PHOSPHO1, nSMase2 and TNAP is coordinately regulated by continuous PTH exposure in mineralising osteoblast cultures. Calcif Tissue Int. 2016;99(5):510-24. 10.1007/s00223-016-0176-9 PMC5055575 27444010

[17] - Kuroshima S, Kovacic BL, Kozloff KM, McCauley LK, Yamashita J. Intra-oral PTH administration promotes tooth extraction socket healing. J Dent Res. 2013;92(6):553-9. 10.1177/0022034513487558 PMC3654759 23611925

[18] - Gou M, Gong C, Zhang J, Wang X, Wang X, Gu Y, et al. Polymeric matrix for drug delivery: honokiol-loaded PCL-PEG-PCL nanoparticles in PEG-PCL-PEG thermosensitive hydrogel. J Biomed Mater Res A. 2010;93(1):219-26. 10.1002/jbm.a.32546 19557789

[19] - Gong C, Yang B, Qian Z, Zhao X, Wu Q, Qi X, et al. Improving intraperitoneal chemotherapeutic effect and preventing postsurgical adhesions simultaneously with biodegradable micelles. Nanomedicine. 2012;8(6):963-73. 10.1016/j.nano.2011.10.010 22154531

[20] - Fang Y, Wang LP, Du FL, Liu WJ, Ren GL. Effects of insulin-like growth factor I on alveolar bone remodeling in diabetic rats. J Periodontal Res. 2013;48(2):144-50. 10.1111/j.1600-0765.2012.01512.x 22834984

[21] - Liu M, Zhang J, Wang X. A relevant experimental study of alveolar and systemic bone mineral density changes in diabetes rats. Hua Xi Kou Qiang Yi Xue Za Zhi. 2009;27(4):451-4. 19769272

[22] 22 - Ersan N, van Ruijven LJ, Bronckers AL, Olgaç V, Ilgüy D, Everts V. Teriparatide and the treatment of bisphosphonate-related osteonecrosis of the jaw: a rat model. Dentomaxillofac Radiol [Internet]. 2014 [cited 2020 Mar 2];43(1):20130144. Available at:10.1259/dmfr.20130144 PMC3887480 24170800

[23] - Chen H, Fu T, Ma Y, Wu X, Li X, Li X, et al. Intermittent administration of parathyroid hormone ameliorated alveolar bone loss in experimental periodontitis in streptozotocin-induced diabetic rats. Arch Oral Biol. 2017;83:76-84. 10.1016/j.archoralbio.2017.06.033 28732226

[24] - Altan MF, Kanter M, Donmez S, Kartal ME, Buyukbas S. Combination therapy of Nigella sativa and human parathyroid hormone on bone mass, biomechanical behavior and structure in streptozotocin-induced diabetic rats. Acta Histochem. 2007;109(4):304-14. 10.1016/j.acthis.2007.02.006 17395251

[25] - Takahashi S, Kikuchi R, Ambe K, Nakagawa T, Takada S, Ohno T, et al. Lymphangiogenesis and NOS localization in healing process after tooth extraction in akita mouse. Bull Tokyo Dent Coll. 2016;57(3):121-31. 10.2209/tdcpublication.2016-0600 27665690

[26] - Motyl K, Botolin S, Irwin R, Appledorn D, Kadakia T, Amalfitano A, et al. Bone inflammation and altered gene expression with type I diabetes early onset. J Cell Physiol. 2009;218(3):575-83. 10.1002/jcp.21626 19006181

[27] - Li YP, Stashenko P. Proinflammatory cytokines tumor necrosis factor-alpha and IL-6, but not IL-1, down-regulate the osteocalcin gene promoter. J Immunol. 1992;148(3):788-94. 1309841

[28] - Campbell GM, Tiwari S, Hofbauer C, Picke AK, Rauner M, Huber G, et al. Effects of parathyroid hormone on cortical porosity, non-enzymatic glycation and bone tissue mechanics in rats with type 2 diabetes mellitus. Bone. 2016;82:116-21. 10.1016/j.bone.2015.04.049 25952971

[29] - Silhi N. Diabetes and wound healing. J Wound Care. 1998;7(1):47-51. 10.12968/jowc.1998.7.1.47 9510751

[30] - Silva BC, Costa AG, Cusano NE, Kousteni S, Bilezikian JP. Catabolic and anabolic actions of parathyroid hormone on the skeleton. J Endocrinol Invest. 2011;34(10):801-10. 10.3275/7925 PMC4315330 21946081

[31] 31 - Choudhary S, Canalis E, Estus T, Adams D, Pilbeam C. Cyclooxygenase-2 suppresses the anabolic response to PTH infusion in mice. PloS One [Internet]. 2015 [cited 2020 Mar 2];10(3):e0120164. Available at: 10.1371/journal.pone.0120164 PMC4363701 25781979

[32] - Liu GY, Cao GL, Tian FM, Song HP, Yuan LL, Geng LD, et al. Parathyroid hormone (1-34) promotes fracture healing in ovariectomized rats with type 2 diabetes mellitus. Osteoporos Int. 2017;28(10):3043-53. 10.1007/s00198-017-4148-3 28808745

[33] - Kuchler U, Spilka T, Baron K, Tangl S, Watzek G, Gruber R. Intermittent parathyroid hormone fails to stimulate osseointegration in diabetic rats. Clin Oral Implants Res. 2011;22(5):518-23. 10.1111/j.1600-0501.2010.02047.x 21251075

[34] - Wolf M, Jäger A, Abuduwali N, Götz W, Lossdörfer S. Continuous PTH modulates alkaline phosphatase activity in human PDL cells via protein kinase C dependent pathways in vitro. Ann Anat. 2013;195(5):455-60. 10.1016/j.aanat.2013.04.006 23742978

[35] - Zhang L, Balani Y, Trinh S, Kronenberg H, Mu Y. [Differential effects on bone and mesenchymal stem cells caused by intermittent and continuous PTH administration]. Zhonghua Yi Xue Za Zhi. 2018;98(10):781-7. 10.3760/cma.j.issn.0376-2491.2018.10.014 29562406

[36] - Babey M, Wang Y, Kubota T, Fong C, Menendez A, ElAlieh H, et al. Gender-specific differences in the skeletal response to continuous pth in mice lacking the IGF1 receptor in mature osteoblasts. J Bone Miner Res. 2015;30(6):1064-76. 10.1002/jbmr.2433 PMC9045460 25502173

[37] - Smith N. A comparative histological and radiographic study of extraction socket healing in the rat. Aust Dent J. 1974;19(4):250-4. 10.1111/j.1834-7819.1974.tb02789.x 4532503

[38] - Yi J, Mei L, Li X, Zheng W, Li Y, Zhao Z. Effects of continuous and intermittent parathyroid hormone administration on midpalatal suture expansion in rats. Arch Oral Biol. 2019;99:161-8. 10.1016/j.archoralbio.2019.01.014 30710837

